# Development of an Inkjet Setup for Printing and Monitoring Microdroplets

**DOI:** 10.3390/mi13111878

**Published:** 2022-10-31

**Authors:** Beatriz Cavaleiro de Ferreira, Tiago Coutinho, Miguel Ayala Botto, Susana Cardoso

**Affiliations:** 1 INESC Microsistemas e Nanotecnologias, Rua Alves Redol, 9, 1000-029 Lisboa, Portugal; 2 Instituto Superior Técnico, Universidade de Lisboa, 1049-001 Lisboa, Portugal; 3 IDMEC, Instituto Superior Técnico, Universidade de Lisboa, 1049-001 Lisboa, Portugal

**Keywords:** inkjet model, open-source, continuous light visualization, microdroplet tracking, customizable setup

## Abstract

Inkjet printing is a digitally controlled additive technology that allows the precise deposition of droplets. Because it is additive, it enables geometries usually unattainable by other technologies. Because it is digitally controlled, its output is easily modulated, even during operation. Combined with the development of functional materials and their micrometer precision, it can be applicable in a wide range of fields beyond the traditional graphic industry, such as medical diagnosis, electronics manufacturing, and the fabrication of microlenses. In this work, a solution based on open-source hardware and software was implemented instead of choosing a commercial alternative, making the most of inkjet flexibility in terms of inks, substrates, and actuation signal. First, a piezoelectric printhead from MicroFab, driven by an ArduinoDue, was mounted in a 3D printer adapted to ensure precise movement in three dimensions. Then, a monitoring system using a USB digital microscope and a computational algorithm was integrated. Both systems combined allow the printing and measurement of microdroplets by digital regulation of a unipolar signal. Finally, based on a theoretical model and a set of experimentally collected samples, the curve that relates the unipolar signal amplitude to the size of the microdroplets was estimated with an acceptable range of prediction uncertainty.

## 1. Introduction

Additive manufacturing (AM) encompasses technologies that are based on the creation of objects (two or three dimensional) through the deposition of material layer-by-layer. Inkjet printing, in particular, creates precise patterns by depositing consecutive droplets at the micrometer scale. Because it is additive and contactless, it has the ability to deposit material in areas of difficult access, over non-homogeneous surfaces, and to create complex geometries. Moreover, its additive quality makes it environmentally friendly, likely more economic, and requires fewer processing steps. Because it is digitally controlled, it can be easily customized and corrected during operation [1,2]. In recent years, new functional inks and deposition substrates to be used in inkjet techniques have been explored, envisioning novel applications in fields such as medicine, electronics, or optics [3,4,5].

In inkjet printing, the ejection of the droplets is accomplished by applying pressure to a fluid inside the printhead chamber through the nozzle, with the size and velocity of such droplets regulated by the magnitude and evolution of this applied pressure. In the particular case of piezoelectric printheads, this pressure is achieved by expanding and compressing the chamber with a piezo actuator whose deformation, in turn, is regulated by an electrical driving waveform. Therefore, manipulating the droplet characteristics entails manipulating the driving waveform. For piezoelectric printheads, the impact of the driving waveform on the ejected droplet during fly has been widely studied [6,7,8], whereas less focus has been put on the direct relationship between the size of the resulting printed droplet. Reference [9] presents a model that relates the amplitude of the input signal of a solenoid valve that actuates the printhead directly with the diameter of the printed droplets, thus allowing a feedback control of the millimeter width of the printed line in terms of the input signal. Using the fact that both the solenoid valve signal and piezoelectric printheads have a proportional relationship between the amplitude of the actuation signal and the droplet volume, this model will be used and validated on the micrometer scale.

Despite a market existing for the production of inkjet printing equipment, commercial alternatives can be expensive and less prone to customization. Today, the application of open-source solutions in experimental research has proven to be an attractive alternative. Although these solutions do not guarantee the reliability ensured by commercial equipment, they allow the modeling of a system tailored to the specific requirements of the research, enable the readjustment of such a system during the research evolution, and have a more attractive cost [10]. Moreover, the ability to reuse existing equipment according to new needs is specifically convenient given the current equipment shortages and long delivery times associated with commercial solutions, not to mention today’s environmental concerns.

This work focuses on the implementation and characterization of equipment that meets the operating requirements associated with micrometer inkjet printing while keeping it highly customizable. Its major contributions are as follows. First, an accessible and modular solution for accurate inkjet printing mainly based on open-source hardware and software was developed. By using a 3D printer with microstepping drivers, the sought micrometer, and smooth displacement on the three axes is achieved. The use of an Arduino Due connected with a driver circuit for the printhead actuation assures a 1 microsecond resolution when designing the waveform and the required output voltage range without being limited to upfront wave designs or operation configurations that could be fixed on commercial alternatives. Moreover, not only are both equipment, Arduino Due and the 3D printer, easily acquired, but the fact that their code and hardware are known makes it possible to adapt them seamlessly, namely, enabling the integration of sensors or the extraction of information that seems relevant, as well as accepting inputs in the desired format and through convenient channels. The present solution allows both Arduino Due and the 3D printer to be updated from any type of external command through a serial port. The second achievement consists of the implementation of a monitoring system that keeps track of the printing in contrast with the traditional approach of stroboscopic light. To visually monitor the evolution of the printing patterns and evaluate the system performance, a USB microscope and a computational algorithm are integrated, providing real-time information on the printing at the micrometer scale. Finally, a model of the implemented system is presented, allowing the manipulation of the characteristics of the printed droplet as a function of the printhead actuation signal. Knowing that both solenoid valve signal and piezoelectric printheads are controlled with a proportional relationship between the amplitude of the actuation signal and the droplet volume, the model was validated on a tenth of a micrometer scale, which is 100 times smaller than previously reported.

## 2. Physics of Inkjet Printing

The physical concepts underlying the formation of droplets, using a piezoelectric printhead, and their deposition on the substrate are overviewed in this Section.

### 2.1. Droplet Formation: Driving Waveform

**Figure 1:**
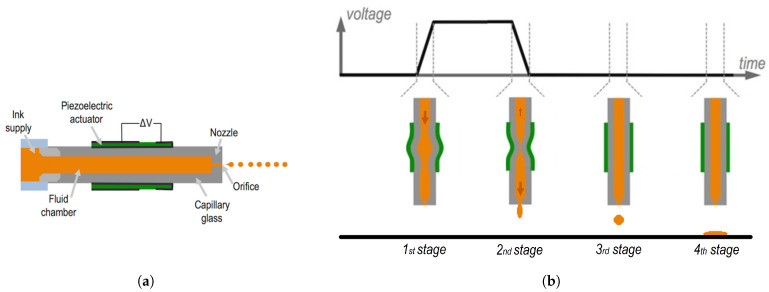
(**a**) Piezoelectric printhead schematic and the (**b**) stages of the formation of a droplet for a unipolar pulse, adapted from [11]. Increasing voltage expands the chamber, pulling the fluid from the reservoir (first stage); then, the decrease in the voltage compresses the chamber, pushing the fluid towards the nozzle (second stage); thus resulting in an ejected droplet (third stage); and finally in a deposited droplet (fourth stage).

Figure 1a shows a tubular printing chamber surrounded by a tubular piezoelectric actuator, with which it moves accordingly. The inner and outer surfaces of the piezo are each bound to an electrode, through which the driving waveform is applied. The fluid chamber is connected to a fluid reservoir on the left side and develops into a small orifice on the right side. The fluid is maintained inside the fluid chamber due to surface tension.

The stages of the formation of a droplet are explained for a unipolar pulse as a driving waveform (Figure 1b). When the voltage increases, the chamber expands along with the piezo, creating a negative pressure inside the fluid chamber. Whereas toward the nozzle, this pressure cannot be propagated, in the opposite direction, it results in the fluid being pulled from the reservoir (first stage). While the voltage is maintained constant, nothing happens apart from the propagation of these pressure waves. When the voltage decreases, the chamber is compressed, thus creating a positive pressure inside the fluid chamber (second stage). If well synchronized, the compression and incoming fluid combined may achieve enough energy to overcome the surface tension at the nozzle and eject a droplet (third stage) [11,12].

The resulting droplet will depend on the applied pulse characteristics. The impact of each pulse parameter is detailed below [6,8,13].

*Pulse amplitude* defines how much energy is used to deform the printhead. It has been observed experimentally that the volume and the velocity of the ejected droplets are proportional to the applied voltage [6,7]. Nevertheless, too much energy may lead to ligament formation and the appearance of unwanted satellites.
*Pulse width* specifies how much time is spent between expansion and compression. Ideally, the pressure wave generated by the compression of the piezo must match the reflected pressure wave generated by its expansion. Any mismatch results in less energy being devoted to ejecting the droplet than is actually available. It has been observed that both the droplet volume and velocity have a near quadratic relationship with the pulse width [6,7,14].
*Pulse frequency* does not have a direct impact on drop formation. Its impact may emerge when the printing is fast enough (less than 100 μs between drops, according to [15]) that residual vibrations from previous drops interfere with the dynamics of the subsequent drops.

Although easy to customize by tailoring the driving waveform, within some limits, the resulting drop characteristics highly depend on the used printhead geometry as well as the fluid rheology, mainly surface tension and viscosity [7,16,17].

### 2.2. Droplet Deposition

When the ejected drop reaches the substrate, a transient phase occurs after the impact. The deposit drop spreads and retracts, dissipating energy until it reaches its equilibrium [18,19]. The size of the printed drop depends on the volume of the ejected droplet and the equilibrium contact angle resulting from the fluid and substrate interaction. Equation (1) translates the relationship between the volume of the falling drop and the diameter of the printed drop [20].

(1) $$d=d_{0}{\left(\frac{8}{tan\frac{\theta }{2}\left(3+{\left(tan\frac{\theta }{2}\right)}^{2}\right)}\right)}^{1/3}$$

where $d_{0}$ is the diameter of the falling droplet, *d* is the diameter of the printed droplet, and θ is the contact angle.

### 2.3. Theoretical Model

In this work, the approach described in [9] is used to mimic the behavior of the printed drops as a function of the amplitude of the actuating pulse. As explained in Section 2.1, the actuating pulse amplitude, *A*, has a proportional effect on the droplet volume. Considering that the contact angle, θ, is constant for the same fluid and substrate, then the relationship between printed diameter and applied amplitude becomes:

(2) $$d^{3}=BA+c$$

where *B* and *c* are constant parameters ∈R. The parameters *B* and *c* are estimated in order to draw the curves that best express the observed experimental data from the implemented system.

## 3. Experimental Setup

**Figure 2:**
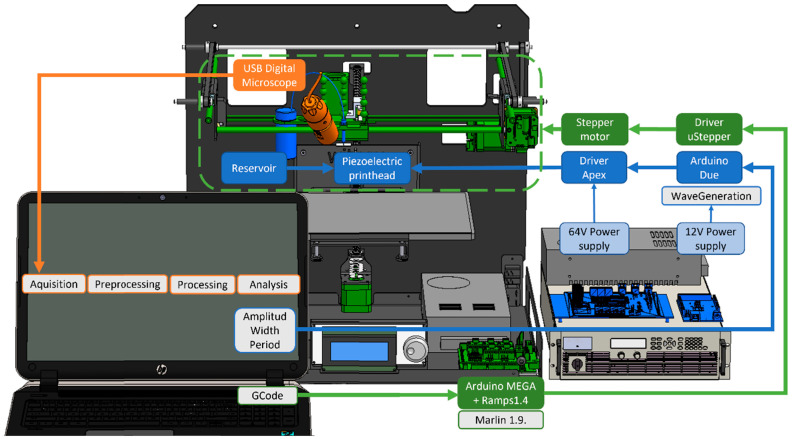
Block diagram of the experimental setup. The information flow and components of the (1) 3D printer, in green; (2) printing system, in blue; (3) and monitoring system, in orange, is displayed.

Two main systems were developed to fulfill different goals. The printing system ensures the ejection of droplets on a micrometer scale. The monitoring system allows the identification and measurement of these printed droplets as they are deposited. In Figure 2, the main elements of the experimental setup that is used are shown. A detailed list of the equipment used can be found in the Supplementary Materials (Table S1 and Figure S1). The programs developed are available at the GitHub repository link (see Supplementary Materials Section).

### 3.1. Printing System

The *printhead* is the main tool for accomplishing microdroplet printing. In this work, a MicroFab MJ-AT-01-050-8MX piezoelectrically actuated printhead (MicroFab, Planto, TX, USA) [21] with a 50 μm orifice is used.

The *drive electronics* to generate the driving waveform are devised to have the ability to modulate an arbitrary driving waveform within a voltage range of [−90; 70] V and a signal resolution of 1μs. The Digital to Analog Converter (DAC) of an Arduino Due (Arduino, Somerville, MA, USA) draws the arbitrary driving waveform, and a driver circuit amplifies this signal to the power required by the printhead. Both Arduino and the driver have their own power supply of 12 and 65 V, respectively. The wire connections between the various components are portrayed in Figures S2 and S3 in the Supplementary Materials.

**Figure 3:**
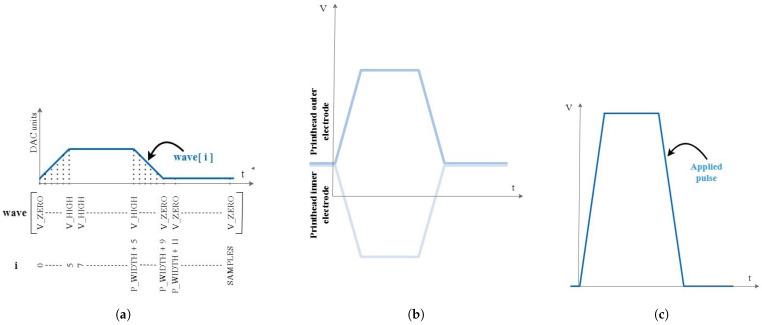
The actuation signal from (**a**) Arduino Due, (**b**) then amplified and shifted with the Apex driver, and (**c**) the actual applied pulse through the piezo.

**Waveform Generation:** Based on the solution presented in [22], the code was adapted to allow implementing changes in the pulse length (“P_WIDTH”), the pulse amplitude (“V_HIGH”), and the pulse period (“samples”) during the printing routine via serial port. The driving waveform update is depicted in Figure 3a. In this study, the unipolar signal is used, but alternative wave shapes were tested with success.

**Signal amplification:** A driver circuit using a double op-amp PA79 (Apex Microtechnology, Tucson, AZ, USA) [23,24] is used to amplify and shift the Arduino Due tailored waveform. The signal applied across the piezo actuator (Figure 3c and Figure S3) is the difference between the two symmetrical amplifications of the Arduino Due signal (Figure 3b). The schematic of the implemented APEX circuit is shown in Figure S4 in the Supplementary Materials.

**Figure 4:**
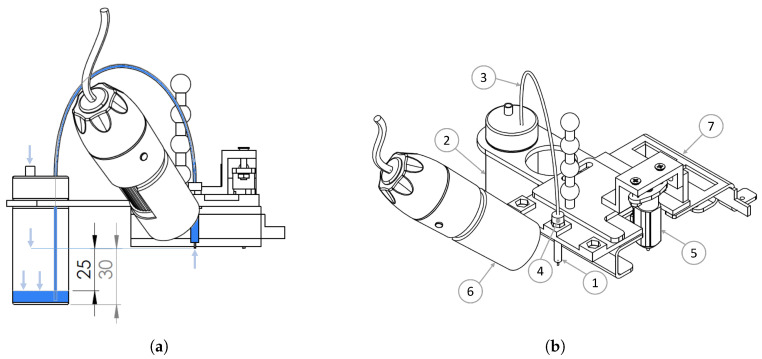
(**a**) Diagram of the fluidic circuit. The arrows represent the atmospheric pressure resulting from venting the reservoir to atmospheric pressure. (**b**) Display of the components mounted on the 3D printer platform (7); the reservoir (2) feeding the printhead (1) through the PTFE tube (3) and the PEEK fitting (4); the camera (6) pointing to the printing area; and lastly the BLTouch (5).

The *fluidic circuit* is responsible for delivering the fluid to the printhead and should be kept at a required operating back pressure to achieve efficient droplet formation. The dispensed fluid used in this project is filtered water and is stored in a 20 mL capacity glass reservoir connected to the printhead by a PTFE tube and PEEK fitting (MicroFab, Planto, TX, USA). The pressure inside the fluidic circuit should guarantee that the fluid is flush at the printhead orifice in equilibrium, which happens with negative hydrostatic pressure, namely [−533; −1600] Pa with respect to the surrounding atmospheric pressure, which is accomplished by venting the reservoir to atmospheric pressure and placing the fluid level inside it between [20; 25] mm underneath the printhead orifice level (Figure 4a).

### 3.2. Monitoring System

A Universal Serial Bus (USB) Jiusion Digital Microscope (Jiusion, Nanjing, China) and Matlab software (The MathWorks, Natick, MA, USA) are used to acquire the droplet images and analyze them.

The *camera* is mounted on the same platform that moves the printhead, as depicted in Figure 4b. To achieve the maximum magnification, the working distance is set at 2.5 cm, resulting in a field of view of 0.9 × 1.2 mm. To correct the geometric distortion, a projection transformation matrix is computed to map every point of the captured frame to the equivalent disposition as if the camera was perpendicular to the printing plane. This matrix is computed by comparing the same grid image, taken on both planes, inclined and perpendicular. With the camera moving, a compromise between speed and sharpness emerges, so the travel speed is limited to 15 mm/min in order to obtain sharp images.

The *algorithm* devised to identify and measure the printed droplets can be divided into four stages: (1) acquisition; (2) preprocessing; (3) processing, and (4) analysis. The acquisition conditions and the computer vision techniques applied in the algorithm are now detailed.

**Figure 5:**
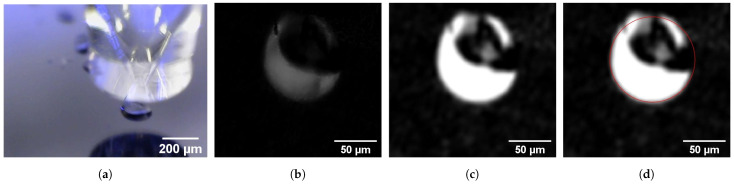
Droplet image evolution: (**a**) Acquisition; (**b**) Preprocessing; (**c**) Processing; (**d**) Analysis.

**Image acquisition:** The Digital Microscope is connected via a serial port and triggered through Matlab with an acquisition frequency of 6 frames per second (fps). The main drawbacks present in the captured images are a reflection on most of the droplet central area and the lack of sharpness due to movement (Figure 5a).

**Image preprocessing:** The captured image is arranged in order to better fit the processes that follow. These arrangements consist of subtracting the image background, converting the image to grayscale, applying the transformation matrix to correct the distortion, and cropping the image to focus on the sub-area containing the printed droplet (Figure 5b).

**Figure 6:**
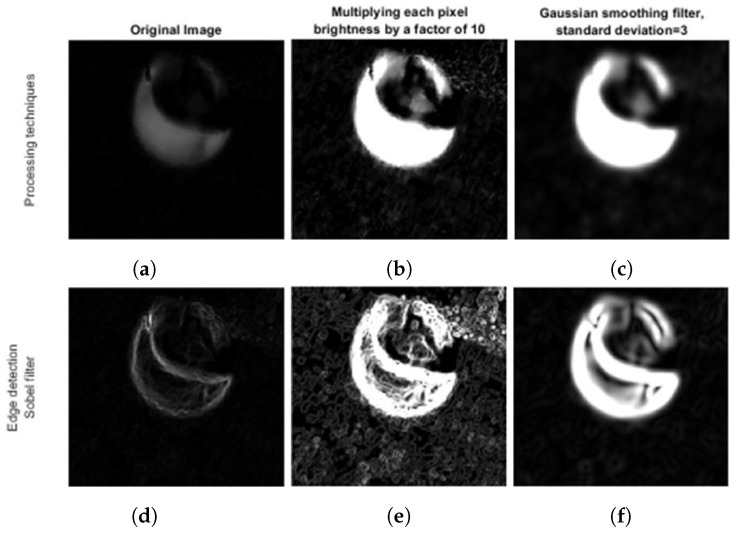
Impact of each processing technique in the edge enhancement of the droplet image when the Sobel filter is applied, used in the CTH algorithm. In the upper row, from left to right, is the droplet image (**a**) after the preprocessing stage followed by (**b**) the brightness enhancement and (**c**) the Gaussian smoothing filter. In the lower row, from left to right, is the image of the droplet after the Sobel filter is applied to the image (**d**) after the preprocessing stage, followed by (**e**) the brightness enhancement and (**f**) the Gaussian smoothing filter.

**Image processing:** The image is processed in order to enhance the droplet for future detection. Each image pixel brightness is multiplied by a factor of 10 to enhance the droplet, followed by a Gaussian filter with a standard deviation of 3 to mitigate the noise (Figure 5c). This proved to be the best combination to correctly identify the droplet in the image analysis stage, with the impact of each technique portrayed in Figure 6.

**Image analysis:** The goal of this analysis phase is to locate the printed droplet and estimate its radius. In order to mitigate the fact that the printed drops do not have perfect geometry, the function *imfindcircles()* [25] is used. The implemented algorithm in such a function is the Circular Hough Transfom (CHT), which looks for gradient arrangements in the image that suggest circular shapes [26], and presents the most likely centers, their radii and their metric (value from 0 to 1, which represents the confidence of each estimation). The three output arrays, radii, centers, and metric, are ordered by descending metric. For each analyzed image, only one droplet is printed, so only the estimated circle with the highest metric is considered a possible droplet candidate (Figure 5d).

In this work, performance is understood as (1) precision, i.e., the low dispersion of the measurements for droplets of the same size; and (2) robustness, i.e., the ability to identify the droplets in less than ideal conditions, such as with poorly cylindrical shape or interrupted edge. The CTH input and output parameter combination that yields the best algorithm performance in detecting and measuring the droplets is considered to be the following:

- Object Polarity: bright. Defines that a possible droplet should be bright on a dark background, eliminating the reflection inside the droplet as a candidate.
- Edge Threshold: 0. Sets the lowest gradient that an edge must have to be considered a possible circle border; a low threshold is used to ensure that the upper edge is less evident due to the reflection inside the droplet and is not dismissed.
- Radius range: previous radius +/−7 pixels. Abrupt changes in the droplet radius are not applied in this work, so the search is set to depend on the last measured drop within a radius interval, reducing the required memory space and time consumption. Nevertheless, this interval should be large enough to avoid the search getting stuck at a local minimum and be able to promptly track changes in the size of the droplet within the variations practiced in our tests.
- Sensitivity: 1. Quantifies how selective the algorithm is about possible candidates for circle centers; a high value is used to not discard less evident or round droplets.

Only results with a metric higher than 0.23 are considered in the data analysis.

### 3.3. Enclosure and Kinematics

A 3D printer, Witbox 1 (BQ, Madrid, Spain), was adapted to mount the printhead and move it along the desired printing trajectory. The platform that accommodates the nozzle moves along the XY plane, while the Z direction sets its distance to the printing bed. The desired trajectory is defined in GCode and sent to the main printer board via a serial port.

One of the main advantages of this choice is the flexibility of adapting the printer’s firmware and hardware to the implemented system requirements, namely:

- Equipment related to plastic deposition is removed, and its configurations are disabled from the printer’s firmware.
- A 3D-printed part is designed to accommodate the printing and monitoring tools, as can be seen in Figure 4b.
- μStepper S drivers (μStepper, Aalborg, Nordjylland, Denmark) are installed in the X and Y stepper motors to achieve smoother displacement and a precision of 1/(80×8) mm/step. They are also used for automatic position correction.
- A Z probe, BLTouch V3.1 (ANTCLABS, Songpa-gu, Seoul, South Korea), is used to correct possible cleavage between the printing bed and the XY plane where the printhead moves.

## 4. Methods

### 4.1. Data Analysis

To identify the system behavior and its dispersion for a given input signal, a set of *n* diameters is measured, and its average and the standard deviation are computed. For each actuating pulse amplitude, $A_{j}$, *n* samples are printed, and the measured diameters, $d_{ij}$, are used to compute the average diameter, $d_{j}$, as seen in (3). This average is considered the accepted “true” diameter for each amplitude.

(3) $$d_{j}=\frac{1}{n}\sum_{i=1}^{n}d_{ij}$$

The standard deviation, $\sigma_{j}$, translates the interval around the average diameter in which its expected, with 68% confidence, that the measured diameters will lie, and it is computed according to (4):

(4) $$\sigma_{j}=\sqrt{\sum \frac{{\left(d_{ij}-d_{j}\right)}^{2}}{\left(n-1\right)}}$$

To quantify the confidence of each average calculation, a weight, $w_{j}$, is computed for each average diameter, $d_{j}$, as the inverse of the standard deviation, $\sigma_{j}$, as seen in (5):

(5) $$w_{j}=\frac{1}{\sigma_{j}}$$

To estimate the parameters of Equation (2), the Matlab *fit()* function with least square formulation and Trust Region method is used with the average diameters weighted by the inverse of its standard deviation as the input data.

### 4.2. Performed Tests

The main goals of this analysis are (1) to identify the monitoring system’s robustness and precision; (2) understand if time or hysteresis affects the experimental data; and (3) estimate the parameters of expression (2) that will allow us to describe the system dynamics. While inkjet printing is generally reproducible, the limitations of the implemented setup and operating conditions may entail otherwise. The main concerns relied on the possibility that (1) a drop in the fluid level during printing may interfere with the drop formation; (2) the range of search based on the previous droplet diameter used in the monitoring algorithm may result in a damped evolution towards the real size after the transition between different amplitudes, and, therefore, influence the average diameter value for such amplitude; and (3) the residual oscillations inside the printhead after an ejected droplet may affect the formation of the next.

Two datasets are collected from operating two tests. The *first test* comprises four sequences of varying actuating pulse amplitude while the remaining inputs are left unchanged. Specifically:

- In the first and third sequences, the amplitude decreases from 3600 to 2500 DAC units in steps of 100 DAC units.
- In the second and fourth sequences, the amplitude increases from 2500 to 3600 DAC units in steps of 100 DAC units.

Therefore, each sequence is composed of 12 intervals of constant actuating pulse amplitude. Each interval is composed of 80 actuating pulses with a period of 1 s. Each actuating pulse has a dwell time of 20 μs and fall and rise times of 5 μs. The fluid level height on the deposit is 0.5 cm, equivalent to 2.5 cm under the printhead orifice.

The *second test* consists of 5200 s of the constant amplitude of 2800 DAC units, with a fluid level height on the deposit of 1 cm.

Both tests use a traveling velocity of the printhead of 15 mm/min, which ensures that the images are well-defined. A 1 s period of the actuating waveform guarantees that at this traveling velocity, the droplets do not fall on top of each other. Temperature, pressure, and humidity conditions are considered constant.

By analyzing the amplitude’s interval dispersion and the second test’s dispersion, we can characterize the system and algorithm uncertainties and reliability. The second test assesses the system time dependence. By comparing sequences with increasing and decreasing amplitude with each other, one can study the dependence in the amplitude gradient sign and detect hysteresis if it exists. By comparing the estimated curves by least squares and the observed data, one can discuss the reliability of the estimated models and their utility as a prediction tool for the implemented system.

## 5. Results and Discussion

The printing and monitoring system’s main limitations are discussed, and the behavior of the joint systems in terms of the diameter of the printed droplets is modulated.

### 5.1. Implemented System Performance

**Figure 7:**
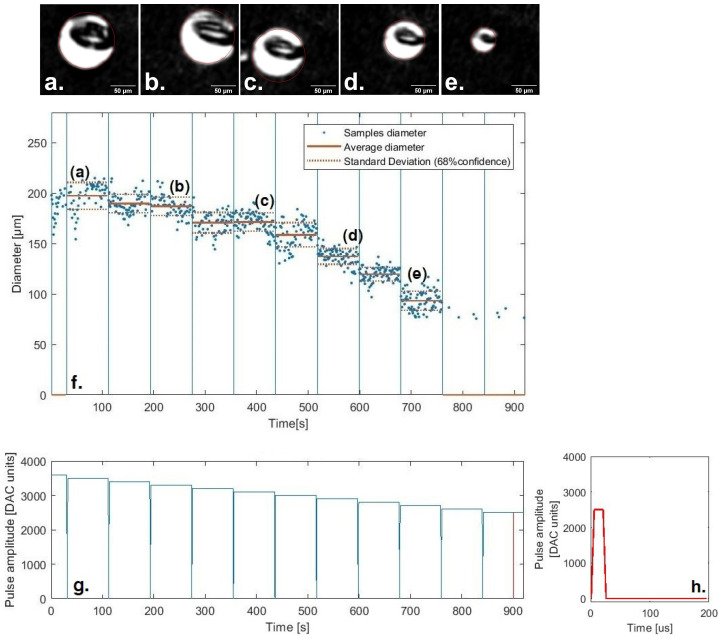
Evolution of the droplet diameter in terms of the amplitude of the actuation pulse. In (**g**) the first sequence of applied amplitudes of the first test is shown, which decreases from 3600 DAC units (0 s) to 2500 DAC units (919 s). In (**f**) the respective average diameters identified by the monitoring system and standard deviation are depicted. (a–e) show examples of the captured droplets for such a sequence of decreasing amplitude, namely, (**a**) 3500 DAC units, (**b**) 3300 DAC units, (**c**) 3100 DAC units, (**d**) 2900 DAC units and (**e**) 2700 DAC units. (**h**) shows an example of an actuation pulse.

Figure 7 shows the average diameters and respective standard deviations for the first amplitude sequence of the first test.

**Figure 8:**
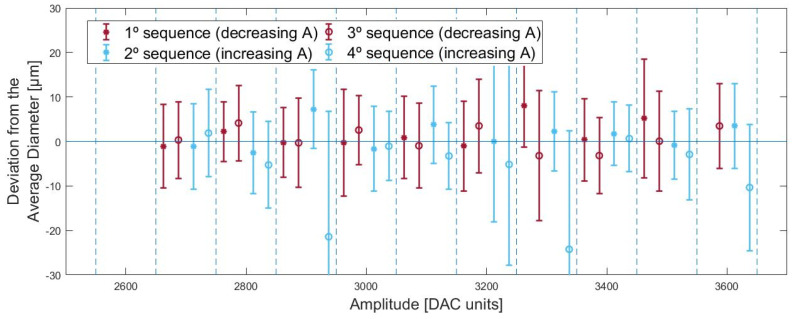
For each amplitude of the first test, the averaged diameters for each sequence are plotted with respect to the overall weighted average.

Apart from some cases, the calculated averages and the standard deviations capture the general behavior of the system and its fluctuations. Figure 8 shows the deviation of the average diameters for each amplitude sequence from the overall weighted average of each applied amplitude. Some measurements, namely in the fourth sequence, presented smaller diameters than what was expected, alongside big standard deviations. These results occurred from the misidentification of smaller circles inside the droplet, which resulted in the calculation of smaller averaged diameters than reality. Such points were later removed from the system dynamics analysis as they did not reflect the system’s behavior. The median of the standard deviations is +/−9.49 μm, which, considering that the drop range is [90; 220]μm, represents an 11% error. This fluctuation appears due to the spreading of droplets caused by insufficient cleaning of the substrate or the inability of the algorithm to identify the exact border. The standard deviation is an estimate of the system fluctuations and does not encompass all the uncertainty that comes from it. Therefore, for a thorough study or stricter applications, such as (1) evaluating the transient system behavior; (2) the impact of temperature or humidity; (3) or to use it as a feedback tool to react to substrate changes in the geometry or wettability; an upgrade to both printing and monitoring system precision would be required. Nevertheless, despite these misidentifications and imprecision, the robustness of the combined systems presents a large dataset from which it is possible to extract with some confidence its average behavior.

### 5.2. Time Invariance

The evolution of the measured printed droplets over a period of 90 min with a constant applied amplitude of 2800 DAC units, which is the dataset resulting from analyzing the second test, is assessed. The average diameters are computed with 80 samples around time instants 320 and 4800 s, as well as the respective standard deviation. By comparing the average diameters at time instants 320 and 4800 s, a slight increase from 123.26 to 127.29 μm along 3480 s can be noted, which can be translated to a $1.16\times 10^{-3}$ μm/s increase. This variation is smaller than any of the estimated standard deviations, thus absorbed by the acceptable range of uncertainty of our measurements, and, therefore, showing that the decrease in fluid in the deposit does not affect the droplet’s size for long printing routines, as is the case of the performed tests (the first test is 3627 s long).

### 5.3. Amplitude Gradient Invariance

Considering that the printing is time-invariant, both increasing amplitude sequences, the second and fourth, are compared with the decreasing amplitude sequences, the first and third, for the first test (Figure 8). No different behavior can be inferred between downward amplitude sequences versus upward amplitude sequences, thus ensuring that the droplet diameter is independent of the amplitude gradient. No damped phenomenon was noticed in the transition between consecutive intervals of different applied amplitudes, reassuring that +/−7 pixels with respect to the previously measured radius is wide enough for the used 100 DAC unit variations. As for residual oscillations, piezo printheads usually work in the order of ms, and in this work, the printhead is actuated with a 1 s period due to the speed limit in order for the camera to capture sharp images. With a 1 s period, it is reasonable to expect that an internal equilibrium between pulses is reached and that each pulse acts as a single pulse. Based on the observed data, it is safe to conclude that for the operating conditions of this test, the system is amplitude gradient invariant. For implementations that allow a higher frequency, such invariance may not hold.

Finally, considering the system time and amplitude gradient invariant, we should conclude that the system is reproducible within our range of precision.

### 5.4. Theoretical Model Validation

**Figure 9:**
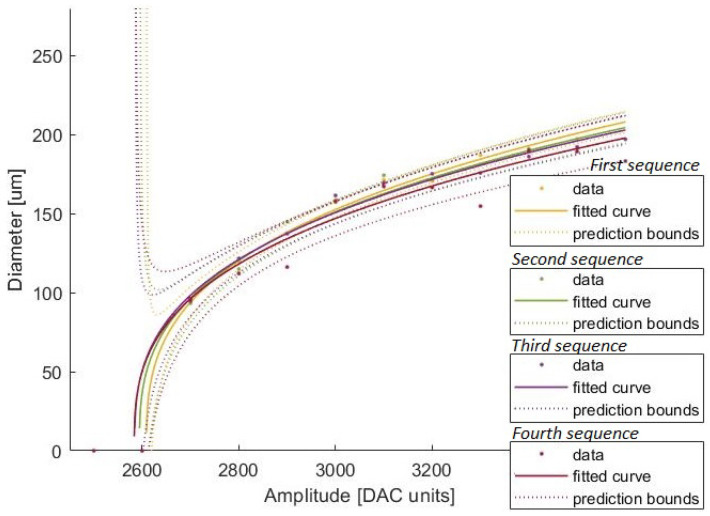
Comparing the estimated curves of all sequences from the first test.

Table 1 shows the estimated parameters with the average diameters for the estimated curves of the first test. The confidence intervals are about 15%, the estimated value of the *B* parameter, except for the fourth sequence, which is 29% of the *B* parameter. This was expected due to the high dispersion of this sequence’s measured diameters. Figure 9 depicts the estimated curves and the average diameters used for its estimation. Apart from the fourth sequence, which is slightly shifted to lower values, all curves lie inside each other’s prediction bounds.

Table 2 presents the predicted diameters calculated from each estimated curve for the highest amplitude from which, experimentally, no drops were printed, 2600 DAC units, and the amplitudes that presented the largest prediction intervals, which correspond to 2700 DAC units and 3600 DAC units. Although experimentally, no droplets were ejected for amplitudes under 2700 DAC units, the predicted diameters from the estimated curves for 2600 DAC units are higher than 0 μm. Nevertheless, such predicted diameters are never higher than 50.54 μm, a size smaller than any measured droplet. This indicates that the lack of droplets under 2600 DAC units amplitude is due to the physical limitations of the system rather than caused by the evolution of its theoretical curve. For 2700 DAC units and 3600 DAC units, the prediction intervals represent an uncertainty between +/−8 and +/−20 μm to estimate the droplet diameter (excluding the fourth sequence). These prediction intervals reveal that the estimated curves are not a precise tool for inferring individual data from them.

The estimated curves present the same performance as the data used to estimate them: although a good tool to reflect the general system behavior, it lacks precision to be useful for a more critical insight into the real dynamics. However, its reflection of the data trend suggests the theoretical model correctly approximates the system’s behavior. A dataset to estimate the curves with smaller spacing between the applied amplitudes would present a more defined system behavior and, therefore, a more complete database for the curve estimation. Future studies combining a less spaced dataset with an improved printing and monitoring system may reveal narrower prediction bounds and so a more accurate representation of the printing system dynamics and its reaction to external factors, such as temperature and humidity.

## 6. Conclusions

In this work, we describe a modular system for printing and monitoring microdroplets. The behavior of this system, namely the relationship between the actuating signal and the diameter of the deposited microdroplets, is modeled through experimental data.

A tailored actuating signal was implemented and tested for printing with great success. We managed to combine the necessary precision of 1 μs with the programming flexibility of an Arduino Due. On the one hand, the flexibility to tailor our waveform with a 1 μs precision allows us to adapt our system to be operated with different inks, substrates, and purposes. On the other hand, the ability to update such a signal in real-time and via a serial port gives us the flexibility to use different software to design and adapt the input signal. A digital microscope connected through USB proves to be effective in capturing images with micrometer resolution, as it moved alongside the printhead, enabling not only a real-time assessment of the printed drops but also the identification and classification of droplets using the CHT algorithm. Nevertheless, such a microscope entails a compromise between precision and traveling velocity. The adaptation of the 3D printer to accommodate both printing and monitoring components allowed a precise displacement and the ability to print accurate patterns and geometries in all three dimensions while using an intuitive and accessible programming language, GCode, as input to define the trajectories. Both printing and monitoring systems combined allowed the printing and measurement of microdroplets with diameters within [90; 220] μm with an uncertainty of +/−9.49μm. The level of precision is appropriate to applications requiring real-time printing monitoring using a low-cost printing setup.

Finally, we implemented a model to correlate the injection input signal amplitude with the diameter of the droplets. Inkjet printing models generally address the droplet size based on the linear relationship between volume and the input amplitude. In this work, we base ourselves on the assumption that the contact angle is constant (for the same liquid and substrate), and thus, the cube of the diameters of the deposited droplets depends linearly on the input amplitude. The theoretical and experimental results are in agreement within a prediction interval of +/−20μm.

Future developments aiming at the improvement of the printing and monitoring system include: (1) implementing an automatic pressure control circuit and studying pressure impact in droplet ejection; (2) implementing a camera with more definition, higher acquisition frequency (fps), and higher shutter speed to freeze the image without compromising the traveling velocity; (3) adding a fluorescent dye to achieve a better contrast between the droplet and the underlying surface; and (4) to use segmentation techniques and morphological operations as the processing techniques, as they are a more precise alternative and are able to detect geometries other than circles. With more accurate and reproducible equipment, the impact of other factors, such as temperature and humidity, could be studied. Finally, having accurate equipment and a complete understanding of its behavior could (1) be used to predict printing routines in terms of inks, patterns, and geometries over which to print or (2) be tested as a feedback tool to react to external changes as substrate variations in geometry or wettability during printing.

## Figures and Tables

**Table 1:** Estimated parameters *B* and *c*.

		First Test		
	1st Sequence	2nd Sequence	3rd Sequence	4th Sequence
**B** **(** μ **m** ${}^{3}$ **/DAC units)**	9091.59	8505.84	8232.72	7914.18
	11%	15%	15%	29%
**c** **(** μ **m** ${}^{3}$ **)**	$-2.40\times 10^{-7}$	$-2.20\times 10^{-7}$	$-2.13\times 10^{-7}$	$-2.04\times 10^{-7}$
	11%	17%	16%	33%

**Table 2:** Predicted diameters, *d*, for applied amplitude of 2600 DAC units and prediction error, *e*, for applied amplitudes of 2700 DAC units and 3600 DAC units.

		First Test		
	1st Sequence	2nd Sequence	3rd Sequence	4th Sequence
**d (2600) (** μ **m)**	42.98	36.53	50.54	50.86
**e (2700) (** μ **m)**	12.43	20.10	17.16	32.75
**e (3600) (** μ **m)**	8.74	12.50	11.62	22.46

## Supplementary Materials

The following supporting information is available at: https://www.mdpi.com/article/10.3390/mi13111878/s1, Table S1: List of the materials used. Figure S1: Experimental setup implemented; Figure S2: Implemented electronics to generate the driving waveform; Figure S3: Applied voltage through the piezoelectric actuator (red) is the difference between the two terminals (yellow–blue); Figure S4: Schematic of the implemented PA79 APEX driver. The programs developed can be downloaded at: https://github.com/MBeatrizCFerreira/Development-of-an-inkjet-setup-for-printing-and-monitoring-microdroplets (accessed on 24 October 2022). Namely, the program for Arduino DUE to generate the actuating waveform; the MATLAB programs that acquires the images from the droplets and analyzes them; and the programs that configure the 3D printer and the μsteppers.

## Abbreviations

The following abbreviations are used in this manuscript:

AM	Additive Manufacturing
DAC	Digital to Analog Converter
USB	Universal Serial Bus
CHT	Circular Hough Transfomer
fps	Frames Per Second
