# Supplementary material for: Development of an Inkjet Setup for Printing and Monitoring Microdroplets
Source: Micromachines (Basel). 2022 Oct 31;13(11):1878. doi: 10.3390/mi13111878 (PMC9694169; doi:10.3390/mi13111878)
Supplement: Supplementary file 1 [file micromachines-13-01878-s001.zip › micromachines-1974614-supplementary.pdf]

micromachines

MDPI

# Supplementary Materials: Development of an inkjet setup for printing and monitoring micro-droplets

**Beatriz Cavaleiro de Ferreira [1,2]*, Tiago Coutinho [1], Miguel Ayala Botto [3] and Susana Cardoso [1,2]**

[1] INESC Microsistemas e Nanotecnologias, Rua Alves Redol, 9, 1000-029 Lisboa, Portugal
[2] Instituto Superior Técnico, Universidade de Lisboa, 1049-001 Lisboa, Portugal
[3] IDMEC, Instituto Superior Técnico, Universidade de Lisboa, 1049-001 Lisboa, Portugal
* Correspondence: beatriz.ferreira@inesc-mn.pt

**Table S1.** List of the materials used.

| No. | Equipment/material | Manufacturer | Available online |
|---|---|---|---|
| 1 | MJ-AT-01-050-8MX printhead | MicroFab, USA | http://www.microfab.com/images/pdfs/manuals/MJ-AT_Manual.pdf |
| 2 | Arduino DUE | Arduino, USA | https://store.arduino.cc/products/arduino-due |
| 3 | PA79 APEX driver | Apex Microtechnology, USA | https://www.apexanalog.com/resources/products/pa79u.pdf |
| 4 | 12V Power Supply | | |
| 5 | 64V Power Supply | | |
| 6 | 2 μStepper S drivers | μStepper, Denmark | https://ustepper.com/store/ustepper-boards/27-ustepper-s.html |
| 7 | Witbox 1 3D printer | (BQ, Spain) | https://www.manua.ls/bq/witbox/manual |
| 8 | BLTouch V3.1 | ANTCLABS, South Korea | https://www.antclabs.com/bltouch-v3 |
| 9 | USB Jiusion Digital Microscope | Jiusion, China | https://www.jiusion.com/products/jiusion |
| 10 | HP ENVY 13-ah0006np laptop | HP, USA | https://support.hp.com/id-en/document/c06047260 |
| 11 | 20 mL capacity glass reservoir | MicroFab, USA | |
| 12 | PTFE tube | MicroFab, USA | http://www.microfab.com/assemblies/ph-47 |
| 13 | PEEK fitting | MicroFab, USA | |
| 14 | Camera and printing mount | (printed on an Ultimaker S3) | |
| 15 | Platform jack | (printed on an Ultimaker S3) | https://www.thingiverse.com/thing:925556 |

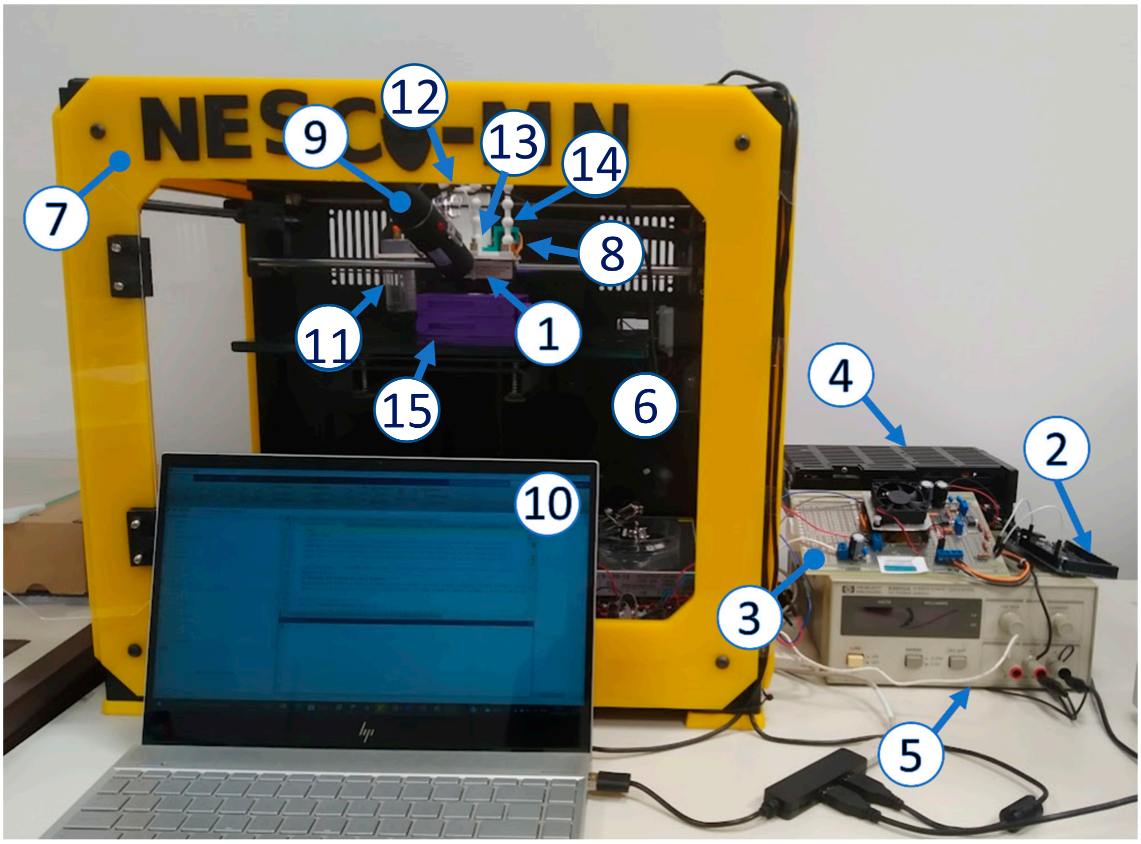


**Figure S1.** Experimental setup implemented.

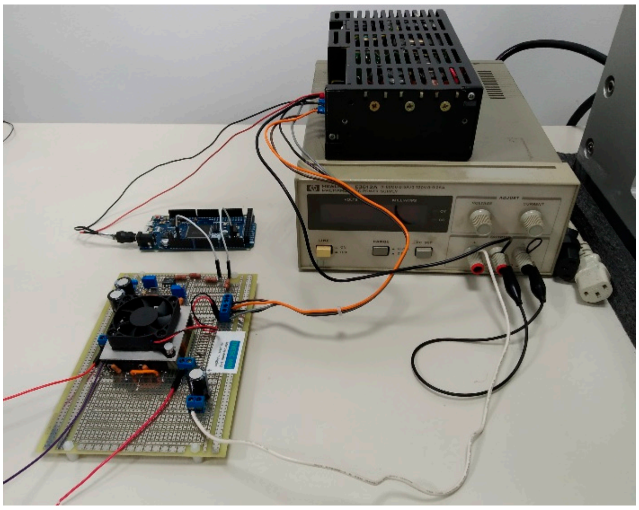

**Figure S2.** Implemented electronic to generate the driving waveform.

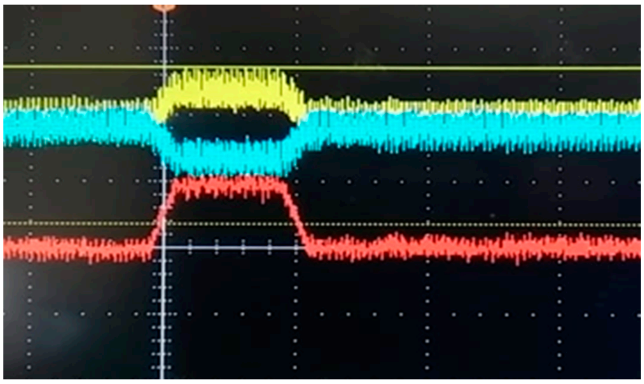

**Figure S3.** Applied voltage through the piezoelectric actuator (red) is the difference between the two terminals (yellow-blue).

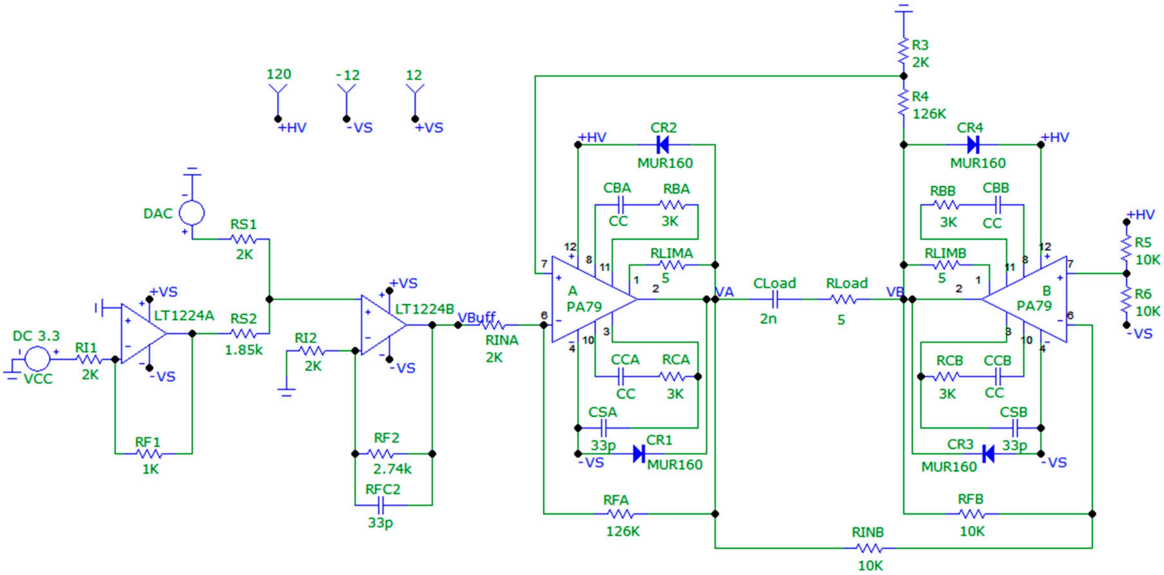


**Figure S4.** Schematic of the implemented PA79 APEX driver. The DC 3.3 VCC input refers to the wire connection with the Arduino DUE 3.3V supply pin and the DAC input refers to the wire connection with the Arduino DUE DAC0 pin.
